# Antiphospholipid Antibody Testing in a Maximum Care Hospital: Method-Dependent Differences

**DOI:** 10.3390/jcm13154528

**Published:** 2024-08-02

**Authors:** Marija Kocijancic, Thomas Goj, Andreas Peter, Reinhild Klein, Sebastian Hörber

**Affiliations:** 1 Institute for Clinical Chemistry and Pathobiochemistry, Department for Diagnostic Laboratory Medicine, University Hospital Tübingen, 72076 Tübingen, Germany; marija.kocijancic@med.uni-tuebingen.de (M.K.); thomas.goj@med.uni-tuebingen.de (T.G.); andreas.peter@med.uni-tuebingen.de (A.P.); 2 Institute of Diabetes Research and Metabolic Diseases of the Helmholtz Centre Munich, 72076 Tübingen, Germany; 3 German Center for Diabetes Research (DZD), München-Neuherberg, 85764 Neuherberg, Germany; 4 Department of Haematology, Oncology, Rheumatology, Immunology, University Hospital Tübingen, 72076 Tübingen, Germany; reinhild.klein@med.uni-tuebingen.de

**Keywords:** antiphospholipid syndrome, antibody assay, anticardiolipin antibodies, anti-beta2 glycoprotein I antibodies

## Abstract

**Background:** Antiphospholipid antibody (aPL) testing is critical for the classification of antiphospholipid syndrome. The 2023 ACR/EULAR classification criteria recommend the use of enzyme-linked immunosorbent assays (ELISAs) and specific thresholds for aPL positivity. Since non-ELISA methods are increasingly used, we compared and evaluated ELISA and non-ELISA aPL assays in a real-world maximum care hospital setting. **Methods:** Between January 2021 and June 2024, anticardiolipin (aCL; IgG and IgM) and anti-beta2 glycoprotein I (aß2GPI; IgG and IgM) antibodies were measured using ELISA (*n* = 5115) and a chemiluminescence-based automated immunoassay (CLIA) (*n* = 3820). Results of parallel testing were compared, and associations with clinical and laboratory characteristics were evaluated. **Results:** A total of 946 samples were tested using ELISA and CLIA in parallel. A total of 136 (14%) specimens were positive for at least one aPL, and 55 (6%) specimens were from patients diagnosed with APS. Among the latter, 47 (85%) and 41 (75%) patients were positive when ELISA- or CLIA-based aPL assays were used, respectively. After applying the >40 units threshold of the new classification criteria, the number of aPL-positive specimens was significantly lower. In the entire cohort, the agreement between ELISA and CLIA aPL assays was acceptable only for aß2GPI IgG; the results from the two methods did not agree for aCL IgG/IgM and aß2GPI IgM. In APS patients, the agreement between ELISA and CLIA aPL assays was acceptable for aß2GPI IgG and IgM but poor for aCL IgG and IgM. Antibody levels in APS patients were significantly higher using CLIA compared to ELISA. **Conclusions:** The method-dependent discrepancies between ELISA- and CLIA-based aPL assays regarding the quantitative and qualitative results are substantial. Both methods are suitable for APS classification, but the choice of aPL assay may influence the classification, and therefore, aPL results should be interpreted carefully in the clinical context.

## 1. Introduction

Antiphospholipid syndrome (APS) is a systemic autoimmune disorder characterized by the occurrence of vascular thrombosis and/or obstetric complications and is driven by a heterogeneous group of autoantibodies called antiphospholipid antibodies (aPL) [1,2,3]. Antiphospholipid antibodies bind phospholipids and phospholipid-binding proteins on cell surfaces, leading to the activation of platelets, monocytes, neutrophils and endothelial cells. Thus, they activate the circulating intravascular environment towards in situ thrombosis and promote other autoimmune and inflammatory processes [3,4]. Although APS is currently considered a single entity, the clinical and biological features of the vascular involvement differ significantly from those associated with obstetric complications [4,5]. APS may be primary when occurring separately or secondary when associated with other autoimmune diseases. Another rare form of APS is catastrophic antiphospholipid syndrome (CAPS), which is characterized by a severe clinical picture of multiple thromboses involving mainly small vessels [6,7].

Until recently, the classification of APS was based on the Sapporo criteria published in 1999 and revised with the 2006 Sydney criteria [8,9]. Accordingly, APS was defined when at least one clinical criterion (thrombotic event and/or obstetric morbidity) and at least one laboratory criterion (persistently positive lupus anticoagulant (LA) and/or persistently positive anticardiolipin and/or anti-beta2 glycoprotein I IgG/IgM) were present. Given the “limitations” of the Sapporo/Sydney criteria, new classification criteria have been approved by the American College of Rheumatology (ACR) Board of Directors and the European Alliance of Associations for Rheumatology (EULAR) Executive Committee [10]. The new criteria were developed primarily for use in clinical observational studies and trials and cover both the clinical and laboratory aspects of APS. The new classification is based on a scoring system that includes six clinical (macrovascular venous thromboembolism, macrovascular arterial thrombosis, microvascular, obstetric, cardiac valve and hematology) and two laboratory (aPL test using a coagulation-based functional assay and a solid-phase-based assay) domains. For the first time, specific recommendations for the laboratory are given, stating that antiphospholipid antibody testing is performed using solid-phase methods. It is recommended that only ELISA methods for anticardiolipin IgG and IgM and anti-beta2 glycoprotein I IgG and IgM should be used [10]. In addition, the use of specific thresholds such as moderate (40–79 units) and high (>80 units) is recommended by the novel classification criteria. However, ELISA tests have been replaced in many laboratories by other methods such as fluorescence enzyme immunoassay (FEIA), chemiluminescence immunoassay (CLIA) and multiplex flow immunoassay (MFI), which have shown improved analytical performance [11,12].

Therefore, the aim of the present study was to compare the diagnostic performance of IgG and IgM aCL and aβ2GPI antibody assays using ELISA and CLIA methods and to evaluate the impact of the 2023 ACR/EULAR classification criteria on aPL positivity in samples from patients in a maximum care hospital.

## 2. Materials and Methods

### 2.1. Data Collection

**Figure 1:**
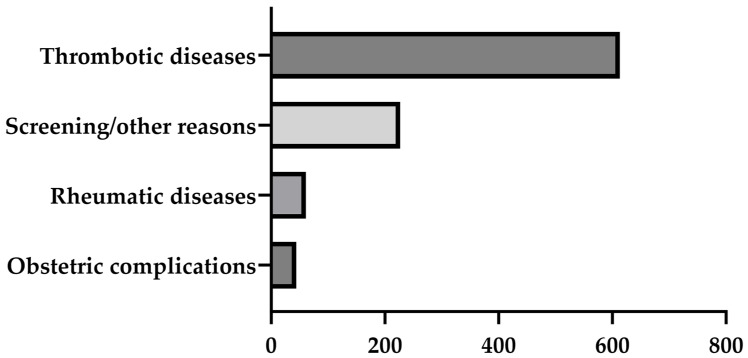
Reasons for antiphospholipid antibody testing in the current study.

We retrospectively evaluated the results of antiphospholipid antibody (aPL) measurements performed at the Department of Diagnostic Laboratory Medicine at the University Hospital of Tübingen, Germany, between January 2021 and June 2024. The University Hospital of Tübingen is a maximum care hospital with approximately 400,000 outpatient visits and 69,000 inpatient visits per year. Anthropometric, clinical and laboratory data were obtained from medical records. Figure 1 shows the most common reasons for aPL testing. APS was diagnosed according to the Sydney classification and the International Society on Thrombosis and Haemostasis (ISTH) laboratory criteria [8,13]. Analysis and interpretation of aPL results were performed in a completely anonymous manner. The analysis was conducted as part of a diagnostic evaluation study in accordance with the Declaration of Helsinki of 1964 and its subsequent amendments.

### 2.2. Laboratory Assays

Determination of aPL included measurements of anticardiolipin antibodies (IgG/IgM) and anti-beta2 glycoprotein I antibodies (IgG/IgM) using a manual ELISA from Diagnostik-a (Ebringen, Germany) and a CLIA-based anticardiolipin IgG/IgM and anti-beta2 glycoprotein I IgG/IgM immunoassay on a fully automated analyzer (IDS-iSYS) from IDS (Immunodiagnosticsystems, Boldon, UK). Clinicians could choose to order either ELISA-based or CLIA-based aPL assays, or both. Results of antibody measurements were expressed as GPL/MPL-U/mL for anti-cardiolipin immunoglobulins and AU/mL (arbitrary unit) for anti-beta2 glycoprotein I immunoglobulins and evaluated according to manufacturer’s cutoffs (see Table 1). The measuring range of all ELISA-based aPL assays is 2–100 GPL/MPL-U. The measuring ranges for CLIA-based aPL assays is as follows: aCL IgG: 0–640 GPL-U/mL; aCL IgM: 0–300 MPL-U/mL; aß2GPI IgG: 0–867 AU/mL; and aß2GPI IgM: 0–300 AU/mL.

Lupus anticoagulant (LA) was determined using two parallel test systems, including a dilute Russell’s viper venom time (dRVVT) and an aPTT-based approach on Atellica COAG 360 analyzers (all reagents and instruments were from Siemens Healthineers). The test-specific screen-mix and algorithm confirmation were performed and evaluated according to the recommendations of the guidance from the ISTH [13]. Commercially available normal pool plasma (Technoclone, Vienna, Austria) was used for mixing studies. All laboratory procedures were performed according to the manufacturer’s instructions.

### 2.3. Statistical Analysis

Data are presented as medians and interquartile ranges for quantitative variables and as numerical values and percentages for categorical data. Qualitative results were compared using 2 × 2 contingency tables and Cohen’s kappa to evaluate agreement between ELISA and CLIA aPL assays. Quantitative antiphospholipid antibody results were compared using the nonparametric Wilcoxon rank-sum test. A *p*-value < 0.05 was considered statistically significant. Analyses were performed using Analyse-it 5.40 software for Microsoft Excel (Analyse-it Software, Ltd., Leeds, UK) and JMP 16.2.0 software (SAS Institute, Cary, NC, USA).

## 3. Results

**Figure 2:**
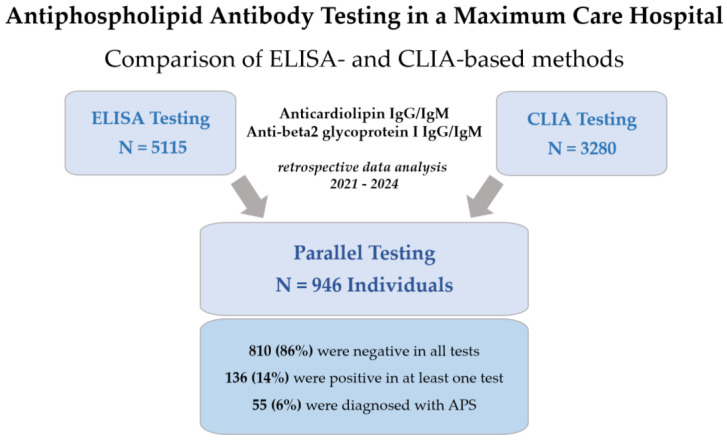
Overview of antiphospholipid antibody testing in the current study.

A total of 8395 samples were tested for antiphospholipid antibodies between January 2021 and June 2024. A total of 5115 samples were measured using ELISA and 3280 using CLIA. Samples from 946 individuals were tested in parallel using ELISA and CLIA (see Figure 2). Among the 946 individuals, the median age was 51 years [interquartile range: 35–61 years], including samples from 466 (49%) women. Table 2 shows the clinical and laboratory characteristics of the individuals tested for aPL.

In the entire cohort (see Table 2, column A), the most common clinical findings included a history of ischaemic stroke (51%) or a thrombotic event (arterial or venous, 15%). Obstetric complications (4%) and autoimmune diseases (7%) were less common. A total of 55 (6%) patients were diagnosed with APS, of which 38 (4%) were classified as primary APS and 17 (2%) as secondary APS. A total of 46 (5%) samples tested positive for lupus anticoagulant (LA), and aPL positivity was observed between 6% (anti-beta2 glycoprotein I IgG) and 10% (anticardiolipin IgM) of all individuals, according to the manufacturers’ cutoffs and independent of the method.

A total of 810 (86%) individuals were negative after all aPL tests were performed (column B). Clinical findings were distributed similarly within the overall cohort. Four APS patients were negative after the ELISA and CLIA aPL assays but had persistently positive LA tests. An additional 22 individuals had a positive LA test but did not meet the criteria for APS classification.

In contrast to the aPL-negative individuals, 136 individuals were positive after at least one aPL test (column C). Among these, the proportion of women and the number of thrombotic events or ischaemic strokes were higher than they were in the entire cohort. Follow-up testing of samples from individuals who were positive after at least one aPL test was performed for 57 (42%) samples.

After comparing the characteristics of the positive ELISA- and CLIA-based aPL assays (columns D and E), the clinical findings were similar. However, the proportion of aPL positivity varied between the ELISA- and CLIA-based aPL results. Consequently, the number of patients with APS differed between the two groups. A total of 47 (51%) patients were positive after at least one ELISA-based aPL assay, and 41 (44%) were positive after at least one CLIA-based aPL assay. After comparing ELISA and CLIA in relation to APS diagnosis, 47 (85%) and 41 (75%) patients were positive using ELISA-based and CLIA-based aPL assays, respectively. In general, positive anticardiolipin IgG results were more frequently observed with ELISA-based aPL assays (49 vs. 30) in contrast to anticardiolipin IgG (49 vs. 63) and anti-beta2 glycoprotein I IgM (24 vs. 65), which were more frequently observed with CLIA-based aPL assays. Anti-beta2 glycoprotein I IgG positivity was similarly distributed between the two methods.

After applying the recommended moderate positivity threshold (>40 units/mL) from the 2023 ACR/EULAR classification criteria, the number of samples meeting this criterion was significantly reduced, depending on the aPL isotype.

### 3.1. Concordances of Antiphospholipid Antibody Assays

#### 3.1.1. Results of APL Measurements in the Entire Cohort

First, aPL measurements were analyzed for the entire cohort. After comparing the qualitative results of the aPL measurements, the ELISA aCL IgG and IgM assays showed a positive percent agreement of 49.0% and 55.1% (IgG: Cohen’s kappa 0.59 [95% confidence interval (CI): 0.46–0.72]; IgM: Cohen’s kappa 0.45 [0.33–0.57]) with CLIA aCL assays, respectively (see Table 3). The analysis using the ELISA aß2GPI IgG and IgM assays revealed a positive percent agreement of 74.2% and 83.3% (IgG: Cohen’s kappa 0.75 [0.62–0.87]; IgM: Cohen’s kappa 0.43 [0.30–0.56]) with the CLIA aß2GPI assays, respectively.

#### 3.1.2. Results of APL Measurements in Patients with APS

Next, we compared the ELISA and CLIA aPL measurements in APS patients (see Table 4). The ELISA aCL IgG and IgM assays exhibited a positive percent agreement of 75.0% and 70.8% (IgG: Cohen’s kappa 0.60 [0.39–0.81]; IgM: Cohen’s kappa 0.45 [0.21–0.69]) with the CLIA aCL assays, respectively. The ELISA aß2GPI IgG and IgM assays revealed a positive percent agreement of 87.5% and 94.1% (IgG: Cohen’s kappa 0.67 [0.48–0.87]; IgM: Cohen’s kappa 0.76 [0.58–0.94]) with the CLIA aß2GPI assays, respectively.

### 3.2. Comparison of Antiphospholipid Antibody Levels between ELISA and CLIA Measurements

**Figure 3:**
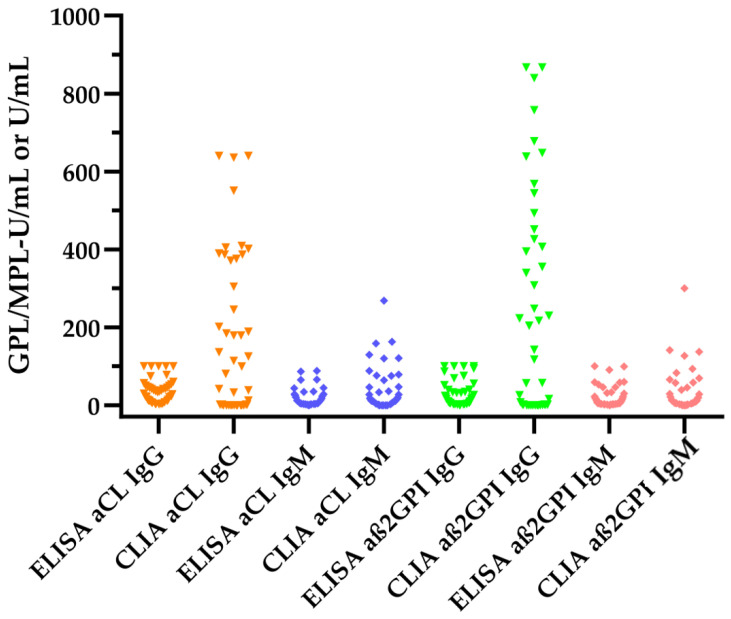
Comparison of antiphospholipid antibody levels between ELISA- and CLIA-based aPL measurements of APS patients. The colours indicate the target antigen: orange, aCL IgG; blue: aCL IgM; green: aß2GPI IgG; red: aß2GPI IgM.

The numerical values of the antiphospholipid antibody results were compared between ELISA- and CLIA-based aPL assays in APS patients. The median values of all aPL measurements were significantly higher using CLIA aPL assays compared to ELISA aPL assays (see Figure 3 and Table 5).

The largest differences were observed for aCL IgG and aß2GPI IgG levels, which were significantly higher for the CLIA measurements compared to the ELISA measurements (aCL: 38 GPL-U/mL vs. 30 GPL-U/mL *p* = 0.0071; aß2GPI: 56 U/mL vs. 10 U/mL, *p* = 0.0003).

## 4. Discussion

In light of the 2023 ACR/EULAR Antiphospholipid Syndrome Classification Criteria, which recommend only ELISA methods for aCL and aß2GPI measurements, including fixed threshold values for positivity, the present study retrospectively investigated the results of antiphospholipid antibody testing in a maximum care hospital.

A significant number of specimens from individuals with a history of thrombotic disease, obstetric complications, autoimmune disease and antiphospholipid syndrome were tested in parallel using commercially available ELISA- and CLIA-based antiphospholipid antibody assays. The results of the comparison showed a considerable degree of variability and low concordance in the results between the assays used, affecting both the classification of a sample as positive or negative and the antibody concentration measured in the samples. Specifically, the results of all aPL measurements in the entire cohort showed low agreement (Cohen’s kappa < 0.60) between the two methods, except for the aß2GPI IgG results (Cohen’s kappa 0.75). Comparisons within the APS cohort showed similar results, with improved agreement for the aß2GPI IgM results (Cohen’s kappa 0.76 vs. 0.43 in the entire cohort).

The results demonstrated in the present study are consistent with several other reports showing discrepancies between different methods for aPL measurements [14,15,16]. However, these discrepancies are not limited to comparisons between ELISA and non-ELISA methods, as inter-assay studies of solid-phase assays have also shown high variability in analytical performance, reported units and agreement regarding quantitative results [14,17]. This can be explained by the detection principle, coating, source of antigens and antibodies, blocking agents to prevent nonspecific binding, dilution protocols, calibration and fixation [17,18]. Moreover, results can vary between methods and manufacturers, and even between batches of the same test system. In contrast, automated test systems, including CLIA-based platforms, demonstrate improved analytical performance with reduced manual handling and lower interlaboratory variability [11,12]. In addition, automated systems can easily handle large sample volumes compared to manual ELISA-based methods. As a result, the number of laboratories using automated aPL testing platforms is increasing, and therefore, ELISA aPL assays are no longer used in the majority of diagnostic laboratories [19].

Despite an increasing number of laboratories using non-ELISA methods for aPL testing, the 2023 ACR/EULAR strongly recommends the use of solid-phase assays for the classification of antiphospholipid syndrome. In addition, the updated classification criteria recommend the use of a semiquantitative interpretation according to thresholds of moderate (40 units) and high (>80 units). Results below the moderate threshold are considered insufficient for APS classification, even in the presence of an appropriate clinical profile. In our study, the use of this threshold would have resulted in a significantly lower number of APS patients (up to 50%), despite their confirmation as APS patients according to the Sydney and ISTH criteria. Consistent with this, Vandevelde et al. showed that the use of 40/80 units as a medium/high threshold is acceptable for aCL/aβ2GPI IgG ELISA but not for CLIA and other non-ELISA methods [14]. To overcome this problem, some studies have attempted to develop CLIA-specific cutoffs that correspond to the moderate/high cutoffs for ELISA aPL assays. In our study, we found that the CLIA aPL results were significantly higher than the ELISA aPL results, which is supported by other studies showing that antibody concentrations in newer automated testing systems are typically significantly higher than those observed with ELISA-based aPL assays [15,20]. Based on these findings, there is currently a debate about the laboratory classification criteria for antiphospholipid syndrome [19,21,22]. It is important to emphasize that the 2023 ACR/EULAR recommendations are aimed at improving the specificity of the classification of APS patients for clinical trials and do not necessarily affect the clinical diagnosis of APS patients. The novel classification guideline takes into account the poor agreement between the numerical values of the ELISA and non-ELISA methods and therefore recommends further validation studies to address the differences between the ELISA and non-ELISA methods for APS classification [10]. Therefore, semiquantitative thresholds should not be used regardless of the method used to detect aPL [21]. Instead, laboratories should follow guidelines such as those of the ISTH Scientific Standardization Committee (ISTH SSC), which recommends that each laboratory use the 99^th^ percentile of a normal population to set decision thresholds for aPL assays, including non-ELISA platforms [13].

Regardless of current guideline recommendations, there is an urgent need to achieve comparability between different aPL assays and platforms. However, a lack of standardization and harmonization is the most significant limitation in APS classification, as there is no universal international reference material for the calibration of aCL and aß2GPI assays. Various reference materials have been developed, such as the Harris or Koike standards [23,24], but none has been universally accepted [18]. Human monoclonal antibodies derived from APS patients may be a good alternative, but it is important to note that patient-derived material may show variability in reactivity from batch to batch. In addition, monoclonal antibodies may not fully represent the reactivity of the patient’s polyclonal antibodies and may not always be detectable by all methods. Because of the inherent differences between assays and results, it is important that samples be tested using the same platform and method for follow-up testing. A recent study defined semiquantitative ranges for ELISA and non-ELISA aPL assays using likelihood ratios [25]. The authors were able to demonstrate improved harmonization between different assay platforms, which may help to establish further aPL assay harmonization programs. Therefore, it will be necessary to conduct large interlaboratory comparison studies utilizing frequently used commercially available ELISA and non-ELISA aPL assays to improve the harmonization process and to achieve a harmonized interpretation of aPL assay results. This may considerably enhance the reproducibility and reliability of the classification and diagnosis of patients with APS.

## 5. Conclusions

This study presents real-world data on antiphospholipid antibody testing in a maximum care hospital. A comparison of ELISA- and CLIA-based aPL assays revealed significant discrepancies in both quantitative and qualitative results. In light of the 2023 ACR/EULAR antiphospholipid syndrome classification criteria, which specifically recommend the use of ELISA-based methods and moderate/high numeric thresholds, our data highlight that method-specific differences should be considered. None of the aPL assays is superior, and therefore, both ELISA and non-ELISA aPL assays are suitable for the laboratory classification of APS patients. However, method-specific decision thresholds should be established, and the results must be carefully evaluated in the clinical context.

## Figures and Tables

**Table 1:** Diagnostic thresholds of antiphospholipid antibody assays used in the current study as reported by manufacturers. Shown are thresholds for positivity.

Antiphospholipid Antibody	ELISA	CLIA
aCL IgG (GPL-U/mL)	>14.4	>20
aCL IgM (MPL-U/mL)	>7.2	>10
aß2GPI IgG (U/mL)	>14.4	>20
aß2GPI IgM (U/mL)	>14.4	>10

Abbreviations: CLIA, chemiluminescent immunoassay; ELISA, enzyme-linked immunosorbent assay; aCL, anticardiolipin; aß2GPI, anti-beta2 glycoprotein I.

**Table 2:** Clinical and laboratory characteristics of individuals who underwent antiphospholipid antibody (aPL) testing.

	A	B	C	D	E
	Entire cohort	All aPL tests negative	Any aPL test positive	Any aPL test positive using ELISA	Any aPL test positive using CLIA
N	946	810	136	92	94
Women	466 (49%)	386 (48%)	80 (59%)	56 (61%)	56 (60%)
Age	51 (35–61)	50 (33–61)	54 (38–65)	51 (36–65)	54 (39–65)
Clinical findings					
Arterial or venous thrombosis	143 (15%)	89 (11%)	54 (40%)	25 (27%)	30 (32%)
Ischemic stroke	482 (51%)	436 (54%)	46 (34%)	29 (32%)	34 (36%)
Transient ischemic attack	40 (4%)	35 (4%)	5 (4%)	3 (3%)	2 (2%)
Obstetric complications	36 (4%)	27 (3%)	9 (7%)	8 (9%)	8 (9%)
Autoimmune diseases					
Connective tissue diseases	36 (4%)	19 (2%)	17 (13%)	16 (17%)	13 (14%)
Vasculitis	21 (2%)	18 (2%)	3 (2%)	2 (2%)	2 (2%)
Others	13 (1%)	7 (1%)	6 (4%)	2 (2%)	6 (6%)
APS diagnosis	55 (6%)	4 (<1%)	51 (38%)	47 (51%)	41 (44%)
Primary APS	38 (4%)	3 (<1%)	35 (26%)	33 (36%)	29 (31%)
Secondary APS	17 (2%)	1 (<1%)	16 (12%)	14 (15%)	12 (13%)
Laboratory findings					
LA (positive)	46 (5%)	22 (3%)	24 (18%)	23 (25%)	20 (21%)
dRVVT (screen) (s)	31 (29–34)	31 (29–34)	33 (29–45)	37 (31–52)	32 (29–47)
aPTT (Actin FS) (s)	24 (23–27)	24 (23–27)	25 (23–27)	26 (23–29)	25 (23–28)
aPTT (Actin FSL) (s)	27 (25–28)	26 (25–28)	27 (25–31)	29 (26–35)	25 (27–31)
Manufacturer thresholds					
aCL IgG (positive)	57 (6%)	-	57 (42%)	49 (53%)	30 (32%)
aCL IgM (positive)	96 (10%)	-	96 (71%)	49 (53%)	63 (67%)
aß2GPI IgG (positive)	54 (6%)	-	54 (40%)	31 (34%)	30 (32%)
aß2GPI IgM (positive)	88 (9%)	-	88 (65%)	24 (26%)	65 (69%)
Moderate threshold (according to 2023 ACR/EULAR criteria)					
aCL IgG (>40 GPL-U/mL)	41 (4%)	-	41 (30%)	20 (22%)	28 (30%)
aCL IgM (>40 MPL-U/mL)	24 (3%)	-	24 (18%)	6 (7%)	20 (21%)
aß2GPI IgG (>40 U/mL)	29 (3%)	-	29 (21%)	10 (11%)	28 (30%)
aß2GPI IgM (>40 U/mL)	25 (3%)	-	25 (18%)	12 (13%)	21 (22%)

Abbreviations: APS, antiphospholipid syndrome; aPL, antiphospholipid antibody; CLIA, chemiluminescent immunoassay; ELISA, enzyme-linked immunosorbent assay; aCL, anticardiolipin; aß2GPI, anti-beta2 glycoprotein I; LA, lupus anticoagulant; dRVVT, diluted Russell’s viper venom time; aPTT, activated partial thromboplastin time; s, seconds.

**Table 3:** Concordances of antiphospholipid antibody assays in the entire cohort (N = 946).

	CLIA	
ELISA		
aCL IgG	negative	positive
negative	891	25
positive	6	24
ELISA		
aCL IgM	negative	positive
negative	861	22
positive	36	27
ELISA		
aß2GPI I IgG	negative	positive
negative	908	8
positive	7	23
ELISA		
aß2GPI I IgM	negative	positive
negative	877	4
positive	45	20

Abbreviations: CLIA, chemiluminescent immunoassay; ELISA, enzyme-linked immunosorbent assay; aCL, anticardiolipin; aß2GPI, anti-beta2 glycoprotein I.

**Table 4:** Concordances of antiphospholipid antibody assays in patients with APS (*n* = 55).

	CLIA	
ELISA		
aCL IgG	negative	positive
negative	20	3
positive	8	24
ELISA		
aCL IgM	negative	positive
negative	23	8
positive	7	17
ELISA		
aß2GPI I IgG	negative	positive
negative	25	6
positive	3	21
ELISA		
aß2GPI I IgM	negative	positive
negative	33	5
positive	1	16

Abbreviations: CLIA, chemiluminescent immunoassay; ELISA, enzyme-linked immunosorbent assay; aCL, anticardiolipin; aß2GPI, anti-beta2 glycoprotein I.

**Table 5:** Comparison of antiphospholipid antibody levels between ELISA and CLIA.

	ELISA	CLIA	*p*-Value
aCL IgG (GPL-U/mL)	30 (10–47)	38 (0–304)	0.0071
aCL IgM (MPL-U/mL)	6 (3–23)	10 (0–36)	0.0258
aß2GPI IgG (U/mL)	10 (2–34)	56 (0–407)	0.0003
aß2GPI IgM (U/mL)	5 (2–22)	7 (2–29)	0.0022

Abbreviations: CLIA, chemiluminescent immunoassay; ELISA, enzyme-linked immunosorbent assay; aCL, anticardiolipin; aß2GPI, anti-beta2 glycoprotein I.

## Data Availability

The data presented in this study are available on reasonable request from the corresponding author.
